# Insight on Diagnosis and Treatment From Over a Decade of Research Through the University of Chicago Monogenic Diabetes Registry

**DOI:** 10.3389/fcdhc.2021.735548

**Published:** 2021-11-05

**Authors:** Tiana L. Bowden, Lisa R. Letourneau-Freiberg, Balamurugan Kandasamy, May Sanyoura, Persephone Tian, Anastasia G. Harris, Graeme I. Bell, Louis H. Philipson, Rochelle N. Naylor, Siri Atma W. Greeley

**Affiliations:** ^1^ Section of Adult and Pediatric Endocrinology, Diabetes, and Metabolism, Department of Medicine, The University of Chicago, Chicago, IL, United States; ^2^ Section of Adult and Pediatric Endocrinology, Diabetes, and Metabolism, Department of Pediatrics, The University of Chicago, Chicago, IL, United States

**Keywords:** monogenic diabetes, MODY, neonatal diabetes, diabetes mellitus, diabetes registry

## Abstract

Monogenic diabetes is a category of diabetes mellitus caused by a single gene mutation or chromosomal abnormality, usually sub-classified as either neonatal diabetes or maturity-onset diabetes of the young (MODY). Although monogenic diabetes affects up to 3.5% of all patients with diabetes diagnosed before age 30, misdiagnosis and/or improper treatment occurs frequently. The University of Chicago Monogenic Diabetes Registry, established in 2008, offers insight into the diagnosis, treatment, and natural history of individuals known or suspected to have monogenic diabetes. Those interested in participating in the Registry begin by completing a secure web-based registration form found on our website (http://monogenicdiabetes.uchicago.edu/registry/). Participants are then screened for eligibility and consented either by phone, video call, or in person. Relevant medical and family history is collected at baseline and then annually *via* surveys through our secure Research Electronic Data Capture (REDCap) database. The University of Chicago Monogenic Diabetes Registry has enrolled over 3800 participants from over 2000 families. Participants represent all 50 states and more than 20 different countries. To date, over 1100 participants have a known genetic cause of diabetes. While many Registry participants reported being referred through their diabetes care provider (54%), a large portion also learned about the Registry through web searching (24%), friends/family (18%), or other sources (13%). Around two-thirds of those with a known genetic cause had research-based genetic testing completed rather than clinical testing due to insurance coverage difficulties. Of those who were found to have monogenic diabetes, significant delays in diagnosis were identified, which highlights the need for increased access to clinical genetic testing covered by insurance companies specifically within the United States. Among genes that cause a MODY phenotype, *GCK* mutations were the most common (59%) followed by *HNF1A* mutations (28%), while mutations in *KCNJ11* were the most common among genes that cause neonatal diabetes (35%) followed by *INS* (16%). Over the last decade, improvements in data collection for the University of Chicago Monogenic Diabetes Registry have resulted in increased knowledge of the natural history of monogenic diabetes, as well as a better understanding of the most effective treatments. The University of Chicago Monogenic Diabetes Registry serves as a valuable resource that will continue to provide evidence to support improved clinical care and patient outcomes in monogenic diabetes.

## Introduction

Monogenic diabetes is a category of diabetes mellitus caused by single gene mutations or chromosomal abnormalities. Monogenic diabetes can be further classified into neonatal diabetes or maturity-onset diabetes of the young (MODY). Monogenic diabetes comprises as much as 3.5% of all diabetes cases diagnosed before age 30 and there are currently over 30 known genes associated with monogenic diabetes (1–4). Monogenic diabetes is frequently misdiagnosed as either type 1 or type 2 diabetes, and thus improperly treated in most patients. A correct diagnosis of monogenic diabetes is important as it directs disease management and leads to a better understanding of hereditability and long-term outcomes. The University of Chicago Monogenic Diabetes Registry was established in 2008, initially beginning as the first national registry for neonatal diabetes in the United States and later expanding by merging a similar but separate registry for MODY (5, 6).

The University of Chicago Monogenic Diabetes Registry seeks to increase the identification and correct diagnosis of monogenic diabetes cases, and to clarify genotype-phenotype relationships, optimal treatment and long-term outcome for the different genetic causes of monogenic diabetes. The Registry also aims to facilitate recruitment of patients suitable for inclusion in other research studies that will add to the knowledge of the pathophysiology and management of these relatively rare forms of diabetes. Additionally, the Registry strives to improve the care of patients with monogenic diabetes by serving as a source of information for clinicians and researchers, as well as for patients and their families.

## Methods

**Figure 1:**
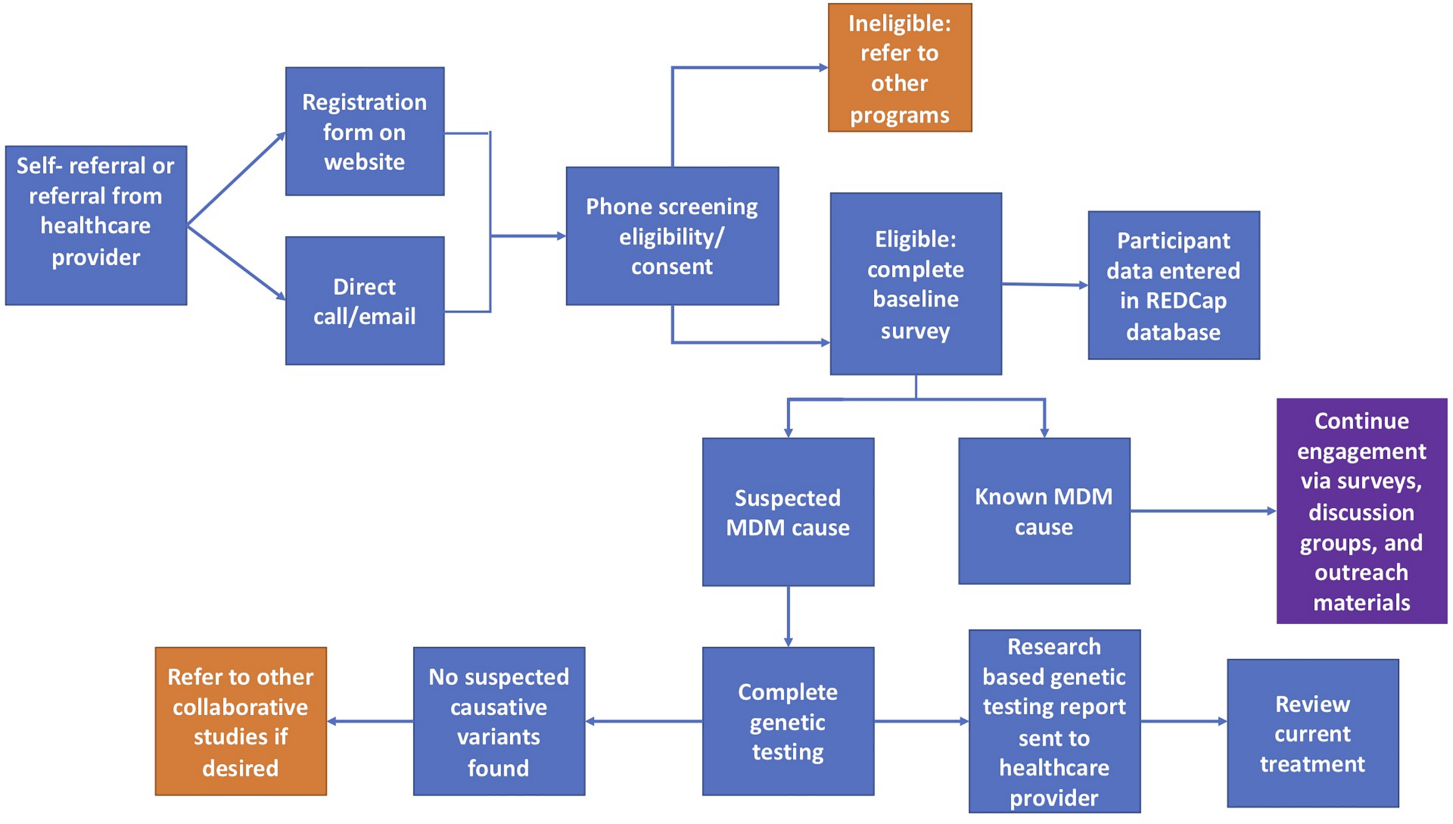
Flowchart of the overall process for participants in the University of Chicago Monogenic Diabetes Registry. Orange squares represent circumstances where other studies or programs are recommended. The purple square represents continued engagement efforts.

Those interested in joining the IRB approved University of Chicago Monogenic Diabetes Registry (UChicago IRB #6858 and #15617B) begin by completing a secure web-based registration form found on our website (http://monogenicdiabetes.uchicago.edu/). This form collects a participant’s basic contact information (name, email, phone number, address), age of diabetes diagnosis, why they suspect monogenic diabetes, provider information, and their referral source. Participants are contacted by a member of the study team, screened for eligibility, and consented either by phone, video call, or in person. During this process, participants are asked to complete either a paper or electronic version of the informed consent form, both of which contain the same information and follow the same regulatory guidelines. Consented participants may also provide a blood or saliva sample for research-based genetic testing (if testing has not yet been completed and insurance coverage for clinical testing is not possible), complete annual surveys, and/or participate in web-based discussion groups (see **Figure 1**). Participants can also choose to be contacted in the future about other studies related to monogenic diabetes.

Our website (http://monogenicdiabetes.uchicago.edu/) provides extensive freely-accessible information about different forms of monogenic diabetes, including features that are suggestive of a monogenic cause of diabetes. Enrollment is open to anyone who is already known to have a monogenic form of diabetes, or who have features that make them more likely to carry an underlying monogenic etiology of their diabetes, such as diabetes diagnosed in infancy (under 13 months of age). Additionally, those with diabetes or hyperglycemia diagnosed after 13 months of age and before 50 years of age with at least one of the following are also eligible: presumed diagnosis of type 1 diabetes with atypical features (negative for diabetes auto-antibodies, well-preserved endogenous insulin production years after diagnosis, or unusual additional syndromic features), presumed diagnosis of type 2 diabetes or gestational diabetes with atypical features (non-obese BMI, lack of signs of insulin resistance, unusual suggestive family history), or incidental diagnosis of mild persistent but non-progressive hyperglycemia without ketosis, and/or pedigree with diabetes present in at least 3 successive generations with a pattern consistent with autosomal dominant inheritance.

The University of Chicago Monogenic Diabetes Registry advises patients and providers on the process for obtaining clinical genetic testing, including providing template letters for prior authorization (these templates are available by emailing monogenicdiabetes@uchicago.edu). Research-based testing is available for participants who are unable to obtain clinical genetic testing related to lack of insurance or coverage restrictions. DNA is predominantly obtained from saliva samples, given that participants are located throughout the US, and occasionally through blood samples. Variants are generally classified according to the American College of Medical Genetics and Genomics (ACMG) five-tier classification system as either: 1) pathogenic, 2) likely pathogenic, 3) uncertain significance, 4) likely benign, or 5) benign (7). For those with likely pathogenic or pathogenic variants, a research report is sent directly to the participant’s provider describing what was found and citing references to any literature that has previously described the variant. Variants of uncertain significance may be reported to the participant’s provider in order to clarify variant pathogenicity by obtaining additional clinical data or family member testing. Cascade genetic testing is recommended either to assist with variant interpretation or to clarify heritability risk. Variants identified by either research-based testing or those obtained through clinical testing may be reanalyzed as needed (for example, when additional family members are tested or new cases are identified) based on current literature, publicly available databases, prediction software, and other case information.

Baseline and longitudinal data regarding demographics, diagnosis, birth history, treatment, family history, and the results of any genetic testing are collected *via* medical records and surveys completed by participants at baseline and then annually. Baseline surveys are sent to participants after they have consented to the study. Invitations to complete annual follow up surveys are automatically emailed to participants a year after their baseline survey or previous follow-up survey was completed. Demographic information such as age, race/ethnicity, education, and income is collected on the baseline survey. Diagnosis information collected on the baseline survey includes the date of diagnosis with hyperglycemia, symptoms, treatment (insulin, sulfonylureas, etc.) and relevant laboratory results from around the time of diagnosis (HbA1c, C-peptide, antibody testing, etc.). Details about participants’ birth history including birth weight, birth length, and gestational age are also collected. Current clinical information, including most recent laboratory values, treatment, other medical problems, and any family history of diabetes is collected on both the baseline and annual follow up surveys. Communication to Registry participants is typically performed by email, phone call, or by mail.

Consistent with Health Insurance Portability and Accountability Act (HIPAA) regulations in the United States, only immediate Registry staff have access to full participant data. Personal identifying information is not shared with outside researchers. All staff members are trained on the importance of participant confidentiality and sign a formal statement agreeing to maintain confidentiality of the data at the time they are employed. As delineated in the consent form, records may be reviewed by federal agencies whose responsibility is to protect human subjects in research including the Food and Drug Administration (FDA) and Office of Human Research Protections (OHRP). In addition, representatives of the University of Chicago, including the Institutional Review Board, may also view the records of the research.

Study data for the Registry are collected and managed using REDCap electronic data capture tools hosted at the University of Chicago (8, 9). REDCap (Research Electronic Data Capture) is a secure, web-based application designed to support data capture for research studies, providing: 1) an intuitive interface for validated data entry; 2) audit trails for tracking data manipulation and export procedures; 3) automated export procedures for seamless data downloads to common statistical packages; and 4) procedures for importing data from external sources. REDCap hosted at the University of Chicago utilizes REDCap LTS Version 11.1.7, PHP 7.3.20 (Linux/Unix OS), and MySQL 8.0.21. REDCap was developed around HIPAA-Security guidelines and is recommended by both the University of Chicago Privacy Office and Institutional Review Board. Only those with a University of Chicago account are allowed access to the system. All web-based information transmission is encrypted and data is stored on virtual machine servers housed on the University of Chicago campus. Servers are physically secured in a continuously staffed and monitored facility and are protected by an enterprise grade firewalls (Palo Alto). Access to the storage system from outside the University of Chicago Campus network is only allowed through the CISCO AnyConnect Secure Mobility Client (VPN software). More information about REDCap can be found at https://www.projectredcap.org.

Web-based discussion groups are available for those with a confirmed diagnosis of either *GCK*-MODY, 6q24-related neonatal diabetes, and *KCNJ11*- or *ABCC8*-related neonatal diabetes, with a plan to form groups for other monogenic diabetes subtypes in the future (10). While our team moderates these groups, conversations between participants are prioritized to build social support.

## Results

**Figure 2:**
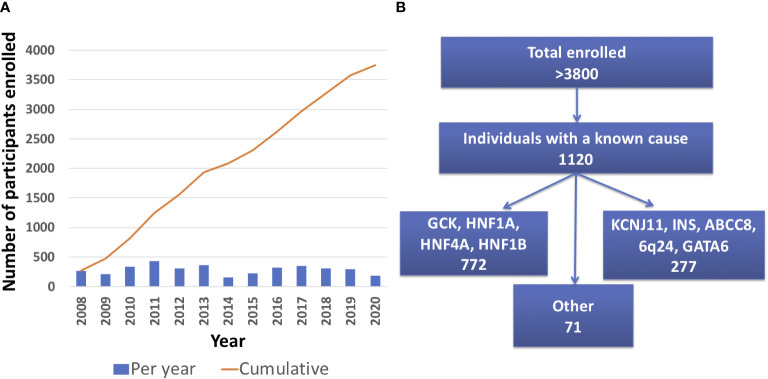
University of Chicago Monogenic Diabetes Registry Enrollment. **(A)** Registry enrollment has steadily increased from 2008 to 2020. **(B)** Over 3800 people have enrolled in the Registry and of those, 1120 have a known genetic cause.

As of April 30^th^ 2021, there were 3834 total participants from 2018 different families enrolled in the University of Chicago Monogenic Diabetes Registry. The Registry has seen an average growth of 288 participants per year since 2008 (**Figure 2A**). Participants represent all 50 states within the United States. There are also participants from over 20 other countries including Canada, Germany, Australia, India, and the Philippines. Most Registry participants report referral by their diabetes care provider (54%), while others credit web searches (24%), friends and family (18%), or other sources (13%).

The majority of Registry participants are female (56%) and self-identify as Non-Hispanic White (84%). Other racial identification includes Black or African American (9%), Asian (9%), American Indian or Alaska Native (2%), Native Hawaiian or other Pacific Islander (<1%), and unknown or preferred not to say (1%). Self-reported ethnicity includes 13% identifying as Hispanic or Latino. Diabetes diagnosis age ranges from birth (age 0) to over 75 years old with a mean diagnosis age of 17 years old (SD 15.9 years).

**Figure 3:**
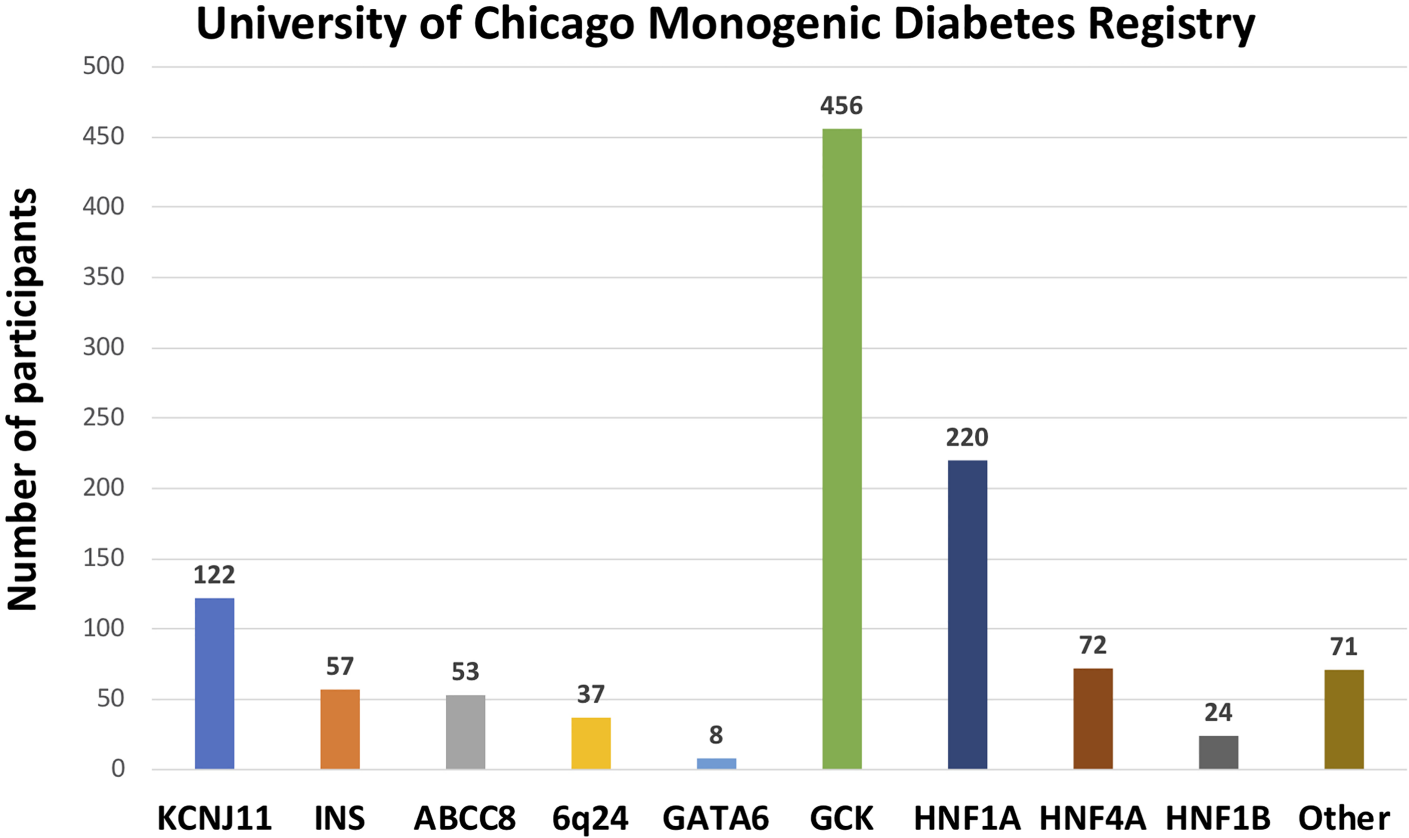
Number of University of Chicago Monogenic Diabetes Registry participants with causative gene variants. GCK variants are the most common found in our Registry, followed by HNF1A, and KCNJ11 variants.

1120 participants have a confirmed genetic cause of diabetes in one of 27 known genes (**Figure 2B**). Of these, 701 participants had research-based genetic testing completed. In the last 6 years, fewer Registry participants had research-based genetic testing completed (59%), compared to the first 6 years of the Registry (71%). The average time to a correct genetic diagnosis from the time of original diagnosis of diabetes was 12.2 years. Among genes that cause a MODY phenotype, *GCK* mutations were the most common (59%) followed by *HNF1A* mutations (28%), while mutations in *KCNJ11* were the most common cause among genes associated with neonatal diabetes (35%), followed by *INS* mutations (16%) (**Figure 3**). From the participants with a known monogenic cause, many were previously told they had type 2 diabetes (26.1%), type 1 diabetes (23.7%), or gestational diabetes (11.8%). 440 participants with MODY (57%) and 108 participants with neonatal diabetes (31%) had a genetic variant that suggested the possibility of a change in treatment.

## Discussion

The University of Chicago Monogenic Diabetes Registry has grown tremendously since its start in 2008. Data from the Registry provides ongoing insight on a variety of aspects of monogenic diabetes including: clinical features that are most suggestive of the possibility of a monogenic cause, clarification of the pathogenicity of gene variants of uncertain significance, and longitudinal data on outcome of different treatments as well as complications. The distribution of monogenic diabetes subtypes found in the Registry is similar to other reports of large cohorts where consanguinity is uncommon (11). Our database includes a large number of participants from across the United States, as well as a small number of participants from other countries. Currently, the great majority of Registry participants identify as Non-Hispanic White, which highlights the need for intentional efforts to increase research participation on monogenic diabetes in underrepresented populations. Topics such as access to genetic testing, provider identification of suspected monogenic diabetes cases, trust of the research community and willingness to participate in research studies, and disclosure of genetic testing results to family members should be further explored across various ethnic and racial groups (12).

Data collection methods for the University of Chicago Monogenic Diabetes Registry have continued to improve over the last decade. The recent introduction of electronic consent forms in addition to paper consent forms has simplified the initial enrollment process for both participants and research staff. Registry consent forms give participants the choice to be re-contacted for future studies related to monogenic diabetes. This option has aided in recruitment efforts for other studies conducted at the University of Chicago Kovler Diabetes Center and at collaborating centers. Registry participants may also be invited to monogenic diabetes family meetings, which is a way for families to connect with each other and learn more about these conditions. While these events are typically held in-person in the Chicago area, the most recent meeting was held virtually over two days in February 2021 due to COVID-19 travel restrictions and was well-attended.

Yearly reminders are sent to participants when it is time for follow-up survey completion, but further study is needed to understand how best to increase long-term retention. As monogenic diabetes represents a diverse group of conditions, future efforts will focus on tailoring data collection for each subtype to improve the relevance of specific data fields and decrease the time required of participants to provide information. Finally, a new participant-facing portal is currently being designed to support improved data collection and encourage participant engagement.

Of Registry participants with a known monogenic cause, the majority received their diagnosis through research-based genetic testing. The majority of participants were referred to the Registry by their diabetes provider, highlighting the barriers faced by clinicians attempting to obtain genetic testing for their patients. Although lack of insurance or payor exclusion of coverage for genetic testing continue to be significant barriers especially within the United States, future efforts are needed to guide clinicians through the many steps to obtaining genetic testing, which has become increasingly available and currently includes lower cost self-pay options as well as direct-to-consumer genetic testing (13). In addition, increased efforts are needed to guide clinicians on most appropriate management recommendations when a monogenic cause is found. Ongoing Registry efforts are aimed at improving the return of results process so that Registry staff can better assist both the provider and participant with proper interpretation of genetic testing reports. Providers and patients are encouraged to reach out to our team *via* email at monogenicdiabetes@uchicago.edu or by phone at 773-702-0829 if they have any questions regarding a monogenic diabetes diagnosis.

## Data Availability Statement

The raw data supporting the conclusions of this article will be made available by the authors, without undue reservation.

## Ethics Statement

The studies involving human participants were reviewed and approved by The University of Chicago Institutional Review Board. Written informed consent to participate in this study was provided by the participants’ legal guardian/next of kin.

## Author Contributions

All authors contributed to either data collection and/or interpretation. LP, GB, SG, and RN are co-Investigators of the study and have contributed to study design and oversight. All authors have reviewed this manuscript and approved the submitted version.

## Funding

This work was supported by the US National Institutes of Health R01DK104942 (Philipson/Greeley), NIH DRTC P30DK020595 (Bell), NIH K23DK094866 (Greeley), NIH CTSA UL1 TR000430 (REDCap support), and gifts from the Kovler Family Foundation.

## Conflict of Interest

The authors declare that the research was conducted in the absence of any commercial or financial relationships that could be construed as a potential conflict of interest.

## Publisher’s Note

All claims expressed in this article are solely those of the authors and do not necessarily represent those of their affiliated organizations, or those of the publisher, the editors and the reviewers. Any product that may be evaluated in this article, or claim that may be made by its manufacturer, is not guaranteed or endorsed by the publisher.
